# Elevated GTP Cyclohydrolase I Pathway in Endothelial Progenitor Cells of Overweight Premenopausal Women

**DOI:** 10.1155/2020/5914916

**Published:** 2020-01-07

**Authors:** Shaohong Wu, Hao He, Ge-Xiu Liu, Xiao-Peng Li, Shun Yao, Huan-Xing Su, Xiang Li, Zi Ren, Haitao Zeng, Jinli Liao

**Affiliations:** ^1^Department of Ultrasound, The First Affiliated Hospital, Sun Yat-sen University, Guangzhou 510080, China; ^2^Department of Cardiology, Nanhai Hospital, Southern Medical University, Foshan 528200, China; ^3^Institute of Hematology, Medical College, Jinan University, Guangzhou 510632, China; ^4^Guangzhou Beijing Community Health Service Center, Guangzhou 510080, China; ^5^Department of Cardiovascular Institute, Guangdong Provincial People's Hospital, Guangdong Academy of Medical Sciences, Guangzhou 510080, China; ^6^State Key Laboratory of Quality Research in Chinese Medicine, Institute of Chinese Medical Sciences, University of Macau, Macau, China; ^7^Department of Ultrasound, Shenzhen Pingle Orthopaedic Hospital, Shenzhen 518000, China; ^8^Center for Reproductive Medicine, The Sixth Affiliated Hospital, Sun Yat-sen University, Guangzhou 510080, China; ^9^Department of Emergency Medicine, The First Affiliated Hospital, Sun Yat-sen University, Guangzhou 510080, China

## Abstract

*Background*/*Aims*. Sexual differences exist in endothelial progenitor cells (EPCs), and various cardiovascular risk factors are associated with the preservation of endothelial function in premenopausal women. However, it is unclear whether differences in endothelial function and circulating EPCs exist between overweight premenopausal women and age-matched men. *Methods*. We compared EPC counting and functions in normal-weight and overweight premenopausal women and men, evaluated endothelial function in each group, and detected the expression of the guanosine triphosphate cyclohydrolase I (GTPCH I) pathway. *Results*. The number of EPCs was lower in the male group than in the female group, regardless of normal-weight or overweight status, and there was no significant difference between the different weight groups among females or males. Endothelial function and EPC migration and proliferation were preserved in overweight premenopausal women compared with overweight men as were nitric oxide (NO) levels in plasma and secreted by EPCs. Endothelial function, the circulating EPC population, and NO levels were not different between normal-weight and overweight premenopausal women. Flow-mediated dilatation was significantly correlated with EPC function, plasma NO levels, and EPC-secreted NO. *Conclusions*. This investigation provides the first evidence for sex-based differences in EPC activity and endothelial function in overweight middle-aged individuals; these differences are associated with alterations in NO production and may partly occur through downregulation of the GTPCH I pathway. The present results provide new insights into the mechanism underlying the preserved endothelial function in overweight premenopausal women and may uncover a potential therapeutic target for endothelial repair in overweight population.

## 1. Introduction

Epidemiological studies have shown an increasing prevalence of overweight and obesity in adults worldwide, resulting in a high incidence of cardiovascular disease (CVD), such as coronary heart disease and peripheral arteriosclerosis, and constituting a major health threat [1, 2]. Body mass index (BMI, calculated as weight in kilograms/(height in meters)^2^), a reasonable and practical estimate of general adiposity, is used routinely in the diagnosis of overweight and obesity. Both increased body weight and increased BMI are correlated with CVD and its risk factors, such as hypertension, diabetes, dyslipidemia, and insulin resistance [3–5]. The established noninvasive flow-mediated endothelium-dependent vasodilatation (FMD) method is widely used to assess endothelial function. Evidence supports the concept that endothelial dysfunction is persistent in overweight and obese individuals, contributing to the pathogenesis and progression of atherosclerotic vascular disease [6–8]. Accordingly, restoration of endothelial dysfunction may be an effective means to maintain vascular homeostasis and prevent obesity-associated cardiovascular complications. Endothelial progenitor cells (EPCs), a group of immature cells derived from the bone marrow, play prominent roles in endothelialization at sites of vascular injury and in the maintenance of endothelial homeostasis, and disorders in EPCs are associated with the progression of atherosclerosis [9–11]. Both the number and the function of EPCs are compromised in the presence of various CVD risk factors, including hyperlipidemia, hypertension, diabetes, and smoking, which may contribute to the progression of endothelial injury and dysfunction [12–14]. A previous study revealed that the numbers of circulating EPCs were reduced in both overweight and obese subjects, with an inverse correlation between EPC counts and BMI [15], and EPC deficits were reversible after significant weight loss [16, 17]. A clinical study further demonstrated that EPC dysfunction is correlated with impaired endothelial function and the associated phenomenon of atherosclerosis in overweight and obesity, suggesting a significant protective effect of EPCs on the endothelium in vivo [18, 19]. Moreover, weight loss can elevate the levels of EPCs in circulating and promote their function, subsequently improving endothelial function [15, 16].

Accumulating evidence has revealed that premenopausal women have a lower risk than postmenopausal women and men of similar age of suffering a broad range of CVDs [20, 21], which is partly associated with the sex-related attenuation of endothelial injury and dysfunction [22, 23]. In healthy middle-aged adults and those with prehypertension, there are sex-based differences in the number or activity of EPCs [24–26], indicating that the alteration of endogenous endothelial repair capacity may conserve endothelial function in premenopausal women. Research has shown that endothelium-dependent vasodilatation is significantly decreased in obese premenopausal women, indicating adiposity-induced endothelial dysfunction in these women [8]. However, it is not clear whether there are sex-based differences in circulating EPCs and endothelial function in overweight middle-aged adults.

Cytokines such as nitric oxide (NO), vascular endothelial growth factor (VEGF), granulocyte-macrophage colony-stimulating factor (GM-CSF), tumor necrosis factor-*α* (TNF-*α*), and interleukin-6 (IL-6) are important factors that regulate circulating EPCs [27–32]. Impairment of endothelium-dependent vasodilatation secondary to decreased NO bioavailability is one of the early deleterious effects of obesity [33], and reduced NO levels may result from increased oxidative stress [34] or proinflammatory cytokine levels, which are predisposing risk factors for CVD. The guanosine triphosphate cyclohydrolase I (GTPCH I)/tetrahydrobiopterin (BH_**4**_) pathway was shown to potentially regulate NO production and impair EPC mobilization and function by endothelial nitric oxide synthase (eNOS) uncoupling in diabetes [35], and it was shown to be involved in declined EPC function in postmenopausal women with overweight [36]. Additionally, VEGF and GM-CSF are potent cytokines that mobilize EPCs from the bone marrow into circulation [29, 30]. Both TNF-*α* and IL-6 are acute inflammatory response mediators that play important roles in systemic inflammation in obesity and are associated with future atherosclerosis [37, 38]; these cytokines have been shown to impair the proliferative, migratory, and tube-forming capacities of EPCs in a dose-dependent manner [31, 32].

Accordingly, we measured EPC number and activity as well as FMD in normal-weight and overweight premenopausal women and men; evaluated the levels of NO, VEGF, GM-CSF, TNF-*α*, and IL-6 circulating in plasma or secreted by EPCs; and investigated the possible underlying mechanism.

## 2. Materials and Methods

### 2.1. Characteristics of Subjects

Twenty overweight premenopausal females (BMI 27.5 ± 2.3 kg/m^2^) and 20 overweight age-matched males (BMI 27.7 ± 2.5 kg/m^2^) were recruited. Forty age-matched subjects of normal weight (20 premenopausal females, BMI 22.6 ± 2.1 kg/m^2^, and 20 males, BMI 23.2 ± 1.5 kg/m^2^) were also included in the control groups. Normal weight was defined as a BMI of 18.5–24.9 kg/m^2^, and overweight was defined as a BMI of over 23 kg/m^2^, according to the weight classifications in the American Heart Association (AHA) Scientific Statement [39]. All enrolled subjects were evaluated through an extensive medical history, routine clinical screening, and laboratory tests to exclude the following statuses and conditions in order to prevent their potential impacts on EPC levels and activity: diabetes mellitus, diagnosed CVD, malignant disease, infection or inflammatory diseases, smoking, irregular menstrual cycles, polycystic ovary syndrome, and previous hysterectomy. Pregnant or breastfeeding women were also excluded. The intake of medications such as antiplatelet, anti-inflammatory, or hypolipidemic agents or sex hormone therapy was not allowed for any of the subjects because these medications have additional effects on circulating EPCs or may weaken gender differences. All the participants refrained from ingesting alcohol or caffeine for 12 h before the study. The experimental protocol was approved by the Ethics Committee of our hospital, and all the participants signed an informed consent form prior to participation in the study.

The baseline characteristics of all the subjects are shown in Table 1. Details regarding peripheral venous blood collection and laboratory tests are provided in a previous study [40].

### 2.2. Evaluation of Circulating EPC Number and Function

EPC counts were evaluated by fluorescence-activated cell sorting (FACS) and cell culture assays as previously described [12, 27, 41, 42]. EPC migratory activity was determined using a modified Boyden chamber, and the proliferative potential was assayed by 3-(4,5-dimethylthiazol-2-yl)-2,5-diphenyltetrazolium bromide (MTT) assays.

### 2.3. FMD Measurement

Brachial artery FMD was measured by high-resolution color ultrasonography with a 5–12 MHz linear transducer on an HDI 5000 system (Washington, USA), as in our previous study and other reports [40, 43]. A baseline image was recorded, and the pressure in an upper-forearm sphygmomanometer cuff was then raised to 250 mmHg for 5 min. The electrocardiogram was monitored continuously for 90 s after cuff deflation. FMD was recorded as the percentage increase in the mean diastolic diameter after reactive hyperemia 55 to 65 s after deflation to baseline.

### 2.4. Measurement of NO, VEGF, GM-CSF, IL-6, and TNF-*α* Levels in Plasma and Secreted by EPCs

NO, VEGF and GM-CSF levels in the plasma and secreted by EPCs were assessed as in our previous study [27]. The plasma concentration of TNF-*α* was evaluated by a high-sensitivity immunoassay, and the IL-6 level was measured by an enzyme-linked immunoassay.

### 2.5. Western Blot Analysis of eNOS and GTPCH I and Measurement of BH_4_

Total protein was harvested from EPCs by cell lysis buffer (Cell Signaling Technology Inc., Danvers, MA, USA). Protein extracts were separated by SDS-PAGE and transferred to polyvinylidene fluoride membranes (Cell Signaling Technology Inc.). Rabbit antiphosphorylated eNOS and anti-eNOS (1 : 1000; Cell Signaling Technology Inc.) and anti-GTPCH I and *β*-actin (1 : 1000; Santa Cruz Biotechnology Inc.) were used to evaluate eNOS and GTPCH I expression as previously described [40, 44, 45]. Intracellular BH_**4**_ concentrations were measured according to previous reports and calculated by subtracting BH_**2**_ plus oxidized biopterin from total biopterins; the results are presented in pmol/mg protein [44, 46, 47].

### 2.6. Statistical Analysis

SPSS V11.0 statistical software (SPSS Inc., Chicago, Illinois) was used for statistical analysis. All data are presented as mean ± SD. Comparisons among the four groups were analyzed by two-factor analysis of variance (sex and weight classification). When indicated by a significant F value, the post hoc Newman–Keuls method was used to identify significant differences among the mean values. Statistical significance was evaluated by ANOVA. Univariate correlations were assessed using Pearson's coefficient (*r*). *P* < 0.05 was considered to indicate statistical significance.

## 3. Results

### 3.1. Baseline Characteristics

The clinical and biochemical characteristics of all the subjects are detailed in Table 1. Height and weight were significantly greater in normal-weight men (167.2 ± 6.1 and 64.8 ± 3.9) and overweight men (168.3 ± 6.5 and 72.1 ± 5.4) than in normal-weight premenopausal women (161.8 ± 5.2 and 59.2 ± 5.8) or overweight premenopausal women (162.8 ± 5.9 and 66.3 ± 6.5) (*P* < 0.05 and *P* < 0.05 for normal-weight and overweight men, respectively, compared to women of the same weight class). Both systolic and diastolic blood pressure were higher in overweight men than in normal-weight men but were equal in the two weight classes of premenopausal women. Estradiol levels were higher in normal-weight and overweight premenopausal women (211.4 ± 33.0 and 200.4 ± 37.6) than in normal-weight or overweight men (97.7 ± 31.3 and 101.9 ± 15.1) (*P* < 0.05 and < 0.05, respectively). FMD was preserved in normal-weight and overweight premenopausal women compared with normal-weight and overweight men (*P* < 0.05 and *P* < 0.05, respectively) but was decreased in overweight men compared with normal-weight men (*P* < 0.05); however, there was no difference in FMD between normal-weight and overweight premenopausal women in our study (*P* > 0.05). There were no differences in blood pressure or in fasting plasma glucose (FPG), low-density lipoprotein (LDL), total cholesterol (TC), triglycerides (TG), or high-density lipoprotein (HDL) levels among the four groups (*P* > 0.05).

### 3.2. EPC Numbers and Activity in the Four Groups

**Figure 1:**
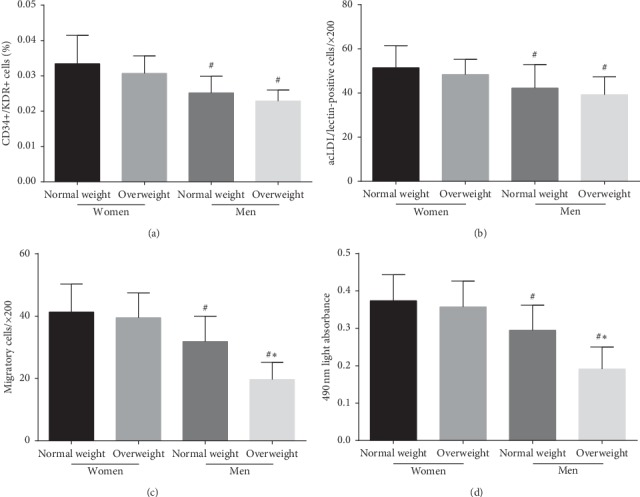
The difference in the number and activity of circulating EPCs in the four groups. Evaluated by (a) FACS analysis and (b) phase-contrast fluorescent microscope, there were no significant difference in the level of circulating EPCs between overweight premenopausal women or men and normal-weight premenopausal women or men. However, the EPC number in normal-weight and overweight men was lower than that in normal-weight and overweight premenopausal women. The migratory (c) and proliferative (d) activity of circulating EPCs in normal-weight and overweight men were lower than those in normal-weight and overweight premenopausal women. There was no difference in the migratory (c) and proliferative (d) activity between normal-weight and overweight premenopausal women. Data are given as mean ± SD. ^*∗*^*P* < 0.05 vs. the same gender group of normal weight; ^*#*^*P* < 0.05 vs. the same weight class of premenopausal women.

The difference in EPC number between normal-weight and overweight individuals was not statistically significant (*P* > 0.05, regardless of sex), as evaluated by FACS analysis and cell culture assays (Figures 1(a) and 1(b)). Both the number and function of EPCs were markedly lower in the male group than in the female group (*P* < 0.05, irrespective of weight classification) (Figures 1(a) and 1(b)). In addition, EPC function was impaired in overweight men compared with normal-weight men but was similar between normal-weight and overweight premenopausal women (Figures 1(c) and 1(d)).

### 3.3. Plasma NO, VEGF, GM-CSF, IL-6, and TNF-*α* Levels in the Four Groups

**Figure 2:**
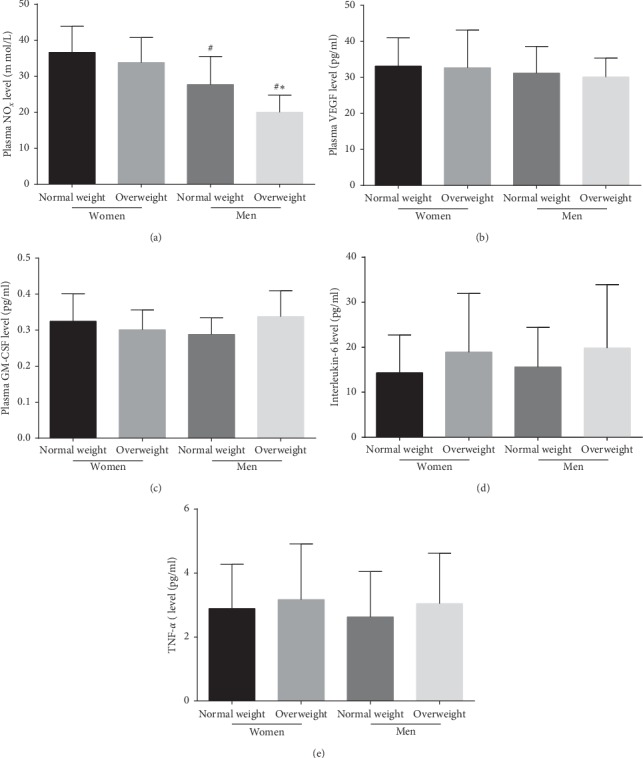
The difference in plasma NO, VEGF, GM-CSF, TNF-*α*, and IL-6 levels in the four groups. (a) The plasma NO level in normal-weight and overweight men was lower than that in normal-weight and overweight premenopausal women. There was no difference in plasma NO level between normal-weight and overweight premenopausal women. No significant difference was found in plasma VEGF (b), GM-CSF (c), TNF-*α* (d), and IL-6 (e) level between the four groups. Data are given as mean ± SD. ^*∗*^*P* < 0.05 vs. the same gender group of normal weight; ^*#*^*P* < 0.05 vs. the same weight class of premenopausal women.

Plasma NO levels were significantly increased in the premenopausal female group compared with the male group (*P* < 0.05 for normal-weight comparison and *P* < 0.05 for overweight comparison) and were higher in normal-weight men than in overweight men (*P* < 0.05). Nevertheless, plasma NO levels were almost equal in normal-weight and overweight premenopausal women (*P* > 0.05). In contrast, the differences in plasma VEGF, GM-CSF, TNF-*α*, and IL-6 levels among the four groups were not statistically significant (*P* > 0.05) (Figures 2(a)–2(c)).

### 3.4. Levels of NO, VEGF, and GM-CSF Secretion by EPCs in the Four Groups

**Figure 3:**
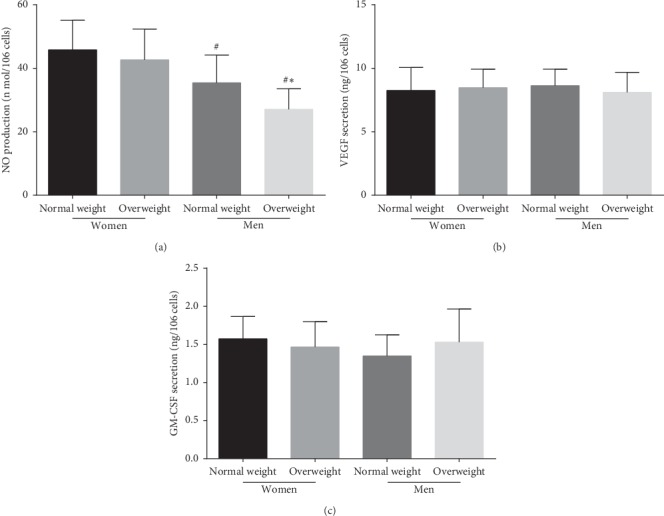
The NO, VEGF, and GM-CSF secretion by EPCs in the four groups. (a) The NO secretion by EPCs in normal-weight and overweight premenopausal women was higher than that in normal-weight and overweight men. There was no difference in NO secretion by EPCs between normal-weight and overweight premenopausal women. (b) No significant difference was found in VEGF secretion by EPCs between the four groups. (c) No significant difference was found in GM-CSF secretion by EPCs between the four groups. Data are given as mean ± SD. ^*∗*^*P* < 0.05 vs. the same gender group of normal weight; ^*#*^*P* < 0.05 vs. the same weight class of premenopausal women.

NO secretion by EPCs was significantly increased in normal-weight and overweight premenopausal women compared with normal-weight and overweight men (*P* < 0.05 and *P* < 0.05, respectively) and was higher in normal-weight men than in overweight men (*P* < 0.05). However, NO secretion was nearly equal between normal-weight and overweight premenopausal women (*P* > 0.05). In contrast, no difference in VEGF or GM-CSF secretion by EPCs was observed among the four groups (Figures 3(a)–3(c)).

### 3.5. Correlation of FMD with EPC Behavior and NO Levels

**Figure 4:**
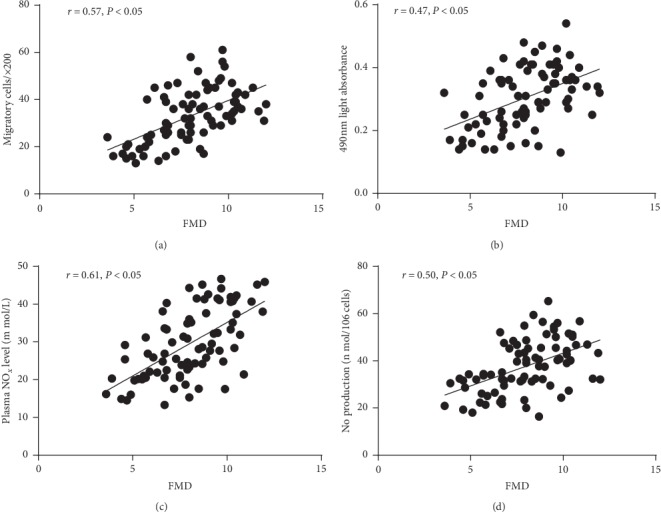
The correlation between circulating EPCs or NO level and FMD. There was a correlation between the EPC proliferatory (a) or migratory (b) and FMD. There was a correlation between the plasma NO level (c) or NO secretion by EPCs (d) and FMD.

Positive correlations between FMD and EPC migratory activity (*r* = 0.65, *P* < 0.05) and proliferative potential (*r* = 0.51, *P* < 0.05) were found in this study (Figures 4(a) and 4(b)). Univariate analysis showed significant correlations between FMD and plasma NO level (*r* = 0.49, *P* < 0.05) and NO secretion by EPCs (*r* = 0.47, *P* < 0.05) (Figures 4(c) and 4(d)).

### 3.6. The GTPCH I/BH_4_ Pathway in EPCs from the Four Groups

**Figure 5:**
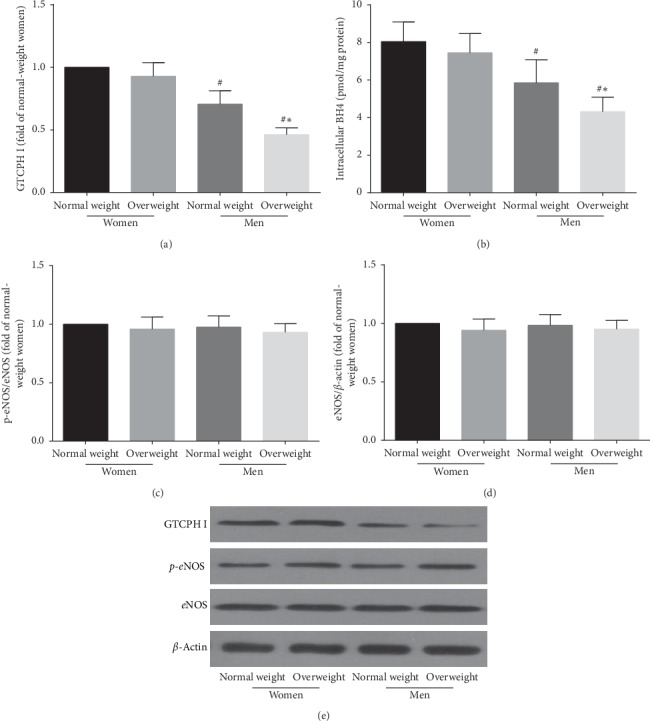
The GTPCH I/BH4 pathway and the phosphorylation of eNOS in circulating EPCs in the four groups. The level of GTPCH I (a) or intracellular BH4 (b) of EPCs in normal-weight and overweight premenopausal women was higher than that in normal-weight and overweight men. There was no difference in the level of GTPCH I (a) or intracellular BH4 (b) of EPCs between normal-weight and overweight premenopausal women. No significant difference was found in either eNOS phosphorylation (c) or eNOS protein expression (d) of EPCs between the four groups. (e) Representative photographs of GTPCH I, phosphorylated eNOS and eNOS expression of EPCs. Data are given as mean ± SD. ^*∗*^*P* < 0.05 vs. the same gender group of normal weight; ^*#*^*P* < 0.05 vs. the same weight class of premenopausal women.

In this study, GTPCH I expression was lower in overweight men than in normal-weight men (*P* < 0.05) but exhibited no difference between the two weight classes of premenopausal women (*P* > 0.05) (Figure 5(a)). The difference in intracellular BH_**4**_ in circulating EPCs between normal-weight and overweight men was statistically significant (*P* < 0.05), but the difference between normal-weight and overweight women was not significant (*P* > 0.05) (Figure 5(b)). In addition, no difference in eNOS expression or phosphorylation was found among the four groups in our study (*P* > 0.05) (Figures 5(c) and 5(d)). These results indicate that the GTPCH I pathway may be a mechanism for the deficiency in NO secretion by EPCs in overweight men, and it likely affects eNOS uncoupling rather than eNOS expression or phosphorylation.

## 4. Discussion

The results of this study showed that EPC migratory activity and proliferative potential were preserved in overweight premenopausal women compared with overweight men, consistent with the alterations in endothelial function. In addition, EPC function was correlated with plasma NO levels and NO secretion by EPCs, indicating that changes in circulating EPCs may be due to varied NO production in overweight middle-aged adults. Müller-Ehmsen et al. [15] analyzed the relationship between BMI and EPC counts in overweight and obese populations and showed that circulating EPC counts in overweight and obese individuals were inversely related to BMI, regardless of sex; furthermore, the reduction in EPC number was reversible upon weight loss in overweight subjects. However, that team did not compare EPC counts between overweight and normal-weight populations or based on gender. Additionally, the study did not exclude smokers or those with hyperlipidemia, diabetes, prior cardiovascular events, or potential medication intake, all of which have been shown to affect EPC levels in peripheral blood. In our study, we found that EPC counts were not significantly lower in overweight subgroups than in normal-weight subgroups (whether female or male), consistent with the results of MacEneaney et al. [17], who found that EPC counts were decreased in obese but not overweight subjects, while the colony-forming capacity of EPCs was impaired in overweight and obese adults compared with normal-weight adults [17]. A previous study found that EPC migratory capacity was not influenced by overweight or obesity [48]. On the other hand, Tsai et al. and Campis et al. revealed that the function of EPCs but not their number is reduced in the context of obesity uncomplicated by atherosclerosis [49, 50]. Our results showed that EPC proliferation and migration were slightly decreased in overweight premenopausal women and men compared with those at a normal weight, but the difference was not statistically significant. Our result suggested a gender difference in FMD in overweight patients, in contrast to the findings of Suh et al. [8], who reported that endothelial function was significantly blunted in obese premenopausal women. A possible reason for the different results may be the difference in the average BMI of the enrolled subjects in the two studies. The BMI range in the previous study was 28.8 ± 3.6 kg/cm^2^, compared with 26.5 ± 1.8 kg/cm^2^ in the female group in our study, and previous research suggests that EPC counts or activity may be correlated with BMI: the higher a person's BMI is, the more obvious the EPC damage [15]. In conclusion, this study suggests a gender difference in the impairment of endothelial function in overweight, which is related to the downregulation of EPC quantity and quality; this study also indicates that enhancing EPC-regulated endogenous endothelial repair capacity is necessary for maintaining endothelial function in overweight men.

Overweight is an intermediate state between normal weight and obesity and confers an increased risk of numerous adiposity-related comorbidities, such as hypertension, diabetes, disrupted lipid metabolism, insulin resistance, and CVD. Therefore, both overweight and obesity are considered the independent risk factors for CVD and are associated with a high risk of CVD morbidity and mortality. Endothelial dysfunction, as a key and early prognostic indicator for atherosclerosis, is responsible for CVD pathogenesis and promotes its progression. Relevant data have shown that endothelial dysfunction, which presents as decreased FMD and is caused by deregulation of the endothelial NO pathway, is persistent in overweight and obesity and is closely related to elevated CVD risk in these weight classes [6–8]. Our recent study found that endothelial function was preserved in premenopausal women with prehypertension [26], indicating the protective effect of the premenopausal state on endothelial function. The current study suggested that FMD was higher in premenopausal women than in men of the same age, regardless of the weight class, and was decreased in overweight men compared to normal-weight men, indicating that endothelial function is attenuated in overweight men. In contrast, there was no difference in FMD between normal-weight and overweight premenopausal women, suggesting that endothelial function is preserved in overweight premenopausal women. The difference in endothelial function between overweight men and premenopausal women may partly explain the reduced risk of CVD in premenopausal women compared to men, suggesting a protective effect of female gender on endothelial function in overweight women and implying that improving endothelial function in overweight individuals may be beneficial for decreasing CVD risk.

Circulating EPCs that are derived from the bone marrow and mobilized into the peripheral blood adhere to the sites of vascular injury and participate in the repair of endothelial injury and angiogenesis. These cells play a vital role in the occurrence and progression of CVD by regulating the endothelial repair capacity in the context of most vascular risk factors. Previous studies have shown that the number or activity of EPCs is decreased in overweight and obese individuals [15–17], and EPC dysfunction impairs endothelial function in the context of overweight and obesity and the associated phenomenon of atherosclerosis [49, 50], suggesting that the decreased endothelial repair capacity is involved in the pathological process of CVD in overweight and obesity. In middle-aged healthy volunteers and those with prehypertension, sex-based differences exist in the number, proliferative potential and migratory ability of circulating EPCs, and these factors are related to the risk of CVD, suggesting that the vascular protection mediated by EPCs in premenopausal women may be due to improved endothelial repair capacity [24–26, 51, 52]. However, it is unknown whether there are similar gender differences in EPCs in overweight middle-aged adults, and the relationship of any such difference with endothelial also remains uncharacterized. In this study, EPC function was decreased in overweight men and was significantly correlated with endothelial function, revealing that EPC impairment in overweight leads to the loss of endothelial repair function and brings about endothelial dysfunction. Nevertheless, the difference in EPCs between overweight and normal-weight premenopausal women was not significant, indicating that gender differences probably play a protective role against vascular damage in overweight women. Furthermore, the present results revealed a positive relationship between endothelial function and EPC activity, indicating that the attenuated endogenous repair capacity of the vascular endothelium mediated by EPCs leads to endothelial dysfunction and confirming the important role of circulating EPCs in maintaining endothelial function in overweight individuals. Increasing the number and function of EPCs may be a vasculoprotective therapeutic strategy for improving endothelial function and preventing the initiation and progression of overweight-associated atherosclerosis.

NO, VEGF, GM-CSF, TNF-*α*, and IL-6 are important for regulating the number and activity of EPC both in healthy subjects and in patients with vascular diseases [27–32], and endogenous NO biosynthesis is required for circulating EPCs to function [53]. In this study, we found that NO levels in plasma and secreted by circulating EPCs were restored in overweight premenopausal women. Furthermore, NO levels in plasma and secreted by circulating EPCs were reduced in overweight men compared with normal-weight men and overweight premenopausal women and paralleled the change in EPC function, highlighting the role of NO in regulating EPC function in overweight middle-aged adults. However, no differences in VEGF, GM-CSF, TNF-*α*, or IL-6 levels were found among the four groups, suggesting that the impaired EPC-mediated endothelial repair capacity in overweight men may be independent of the modulation of these cytokines. In addition, our study found positive correlations between FMD and NO levels, both in plasma and produced by cultured EPCs, suggesting that reduced systemic NO production and endogenous NO biosynthesis by circulating EPCs may contribute to endothelial dysfunction in overweight.

eNOS, a key enzyme in endogenous NO biosynthesis, is constitutively expressed in endothelial cells and involved in the production of NO, which not only modulates the mobilization of EPCs from the bone marrow but also promotes EPC migration and proliferation [28, 53]. eNOS expression and phosphorylation have been shown to be essential for EPC survival, migration, and angiogenesis [54], and the uncoupling of eNOS may result in eNOS-mediated NO production and induce EPC senescence [55]. The subsequent reduction in EPC levels or impairment of EPC function contributes to the pathogenesis of CVD in obesity [18, 19]. Our previous study and other investigations have reported that GTPCH I deactivation results in BH_**4**_ deficiency and subsequent eNOS uncoupling, which may lead to decreased eNOS-mediated NO production; furthermore, the subsequent reduction in EPC levels and the impairment of EPC function likely contribute to the pathogenesis of vascular disease [24, 44]. In this investigation, we found that GTPCH I and intracellular BH_4_ levels were reduced in overweight men compared with overweight premenopausal women and normal-weight men, but no such significant reduction was found in overweight premenopausal women. GTPCH I and BH_4_ were regulated in parallel with the alterations in EPC function and EPC-mediated endothelial function. Meanwhile, eNOS phosphorylation and expression showed differences among the four groups. These results indicated that the GTPCH I pathway may be involved in abnormal NO bioactivity due to eNOS uncoupling but not phosphorylation, leading to a reduction in EPC function and subsequent endothelial dysfunction in overweight people.

The present study has the following implications. First, in overweight men, EPC migration and proliferation are impaired in parallel with attenuated endothelial function. As an independent and valid risk factor for endothelial dysfunction, EPC dysfunction is an early, effective marker of cardiovascular risk in overweight individuals. In addition, early intervention to enhance vascular repair capacity by upregulating EPC function may be an impactful approach to the treatment of overweight-related CVD. Second, we found no difference in FMD or EPCs between normal-weight and overweight premenopausal women in this study, suggesting that premenopausal status exerts a protective effect on EPC and endothelial function in overweight individuals. Third, our study revealed that reduced EPC activity, but not EPC counts, in overweight may contribute to the decreased NO production, at least partly through downregulation of the GTPCH I/BH_4_ pathway. The upregulation of GTPCH I and subsequent improvement in the NO synthesis and plasma levels could enable the modulation of EPC migration and proliferation, thus strengthening the endothelial repair capacity in overweight individuals.

## Figures and Tables

**Table 1:** Clinical and biochemical characteristics.

Characteristics	Normal-weight women	Overweight women	Normal-weight men	Overweight men
	(*n* = 20)	(*n* = 20)	(*n* = 20)	(*n* = 20)
Age (years)	46.4 ± 3.2	47.3 ± 2.78	48.1 ± 4.2	45.9 ± 3.8
Height (cm)	161.8 ± 5.2	162.8 ± 5.9	167.2 ± 6.1^*#*^	168.3 ± 6.5^*#*^
Weight (kg)	59.2 ± 5.8	66.3 ± 6.5^*∗*^	64.8 ± 3.9^*#*^	72.1 ± 5.4^*∗*,*#*^
BMI (kg/cm^2^)	22.6 ± 2.1	27.5 ± 2.3	23.2 ± 1.5	27.7 ± 2.5
Systolic blood pressure (mmHg)	124.5 ± 9.7	148.8 ± 4.1	122.7 ± 5.5	149.3 ± 4.9^*∗*^
Diastolic blood pressure (mmHg)	76.4 ± 7.2	89.3 ± 6.5	75.4 ± 6.7	89.6 ± 5.0^*∗*^
Heart rate (beats/min)	76.8 ± 9.4	78.5 ± 7.5	79.3 ± 8.1	80.7 ± 8.4
AST (mmol/L)	27.2 ± 6.8	25.6 ± 5.1	24.7 ± 6.3	24.6 ± 6.2
ALT (mmol/L)	24.8 ± 7.8	24.0 ± 5.4	22.8 ± 5.1	21.9 ± 5.9
BUN (mmol/L)	5.0 ± 0.9	4.9 ± 1.0	5.2 ± 0.8	5.4 ± 0.9
Cr (mmol/L)	65.6 ± 15.4	63.2 ± 14.5	67.6 ± 15.8	72.5 ± 15.5
LDL (mmol/L)	3.03 ± 0.48	2.89 ± 0.43	2.87 ± 0.43	2.78 ± 0.40
TC (mmol/L)	5.08 ± 0.43	4.83 ± 0.51	4.81 ± 0.65	4.72 ± 0.63
HDL (mmol/L)	1.36 ± 0.26	1.42 ± 0.25	1.38 ± 0.18	1.44 ± 0.21
TG (mmol/L)	1.51 ± 0.19	1.46 ± 0.23	1.44 ± 0.16	1.40 ± 0.22
FPG (mmol/L)	4.55 ± 0.56	4.76 ± 0.51	4.67 ± 0.61	4.36 ± 0.46
Estradiol (pmol/L)	211.4 ± 33.0	200.4 ± 37.6	97.7 ± 31.3^*#*^	101.9 ± 15.1^*#*^
FMD (%)	9.56 ± 1.80	8.61 ± 1.64	8.17 ± 1.759^*#*^	6.51 ± 1.81^*∗*,*#*^

Abbreviation: BMI, body mass index; LDL, low-density lipoprotein; TC, total cholesterol; HDL, high-density lipoprotein; TG, triglyceride; FPG, fasting plasma glucose; hrCRP, hypersensitive C-reactive protein; FMD, flow-mediated brachial artery dilatation. Notes: data are given as mean ± SD. ^*∗*^*P* < 0.05 vs. the same gender group of normal-weight; ^*#*^*P* < 0.05 vs. the same weight class of premenopausal women.

## Data Availability

The data used to support the findings of this study are available from the corresponding author upon resonable request.
